# Periscope of Dermatology

**Published:** 1898-11-26

**Authors:** 


### PERISCOPE OF DERMATOLOGY.

(*Continued from page* 117.)

**Eczema.**—Aubert[6] recommends the treatment of eczema by picric acid. He finds it most useful in acute, and during the exacerbation of chronic cases; it is also useful in calming the itching of chronic eczema, and in impetiginous eczema of children. He uses a watery solution of 12 grains to the litre; compresses soaked in this solution are applied to the parts, care being taken that the evaporation of the liquids is not prevented by the dressing, which is allowed to dry on, and is not interfered with for two or three days. At the end of the treatment, boric vaseline should be applied.

Jacquert[7] recommends the treatment of chronic eczema and psoriasis by repeated scarifications. He first prepares the surface by the more or less prolonged application of potatoe poultices; the surface is then scarified by lines about one or two millimetres apart. and allowed to bleed freely, hot water being applied, The poultices are then reapplied. From six to sixteen sittings are required to cure the disease. Acute eczema is the least amenable to this form of treatment, and lichenoid cases are the most favourable.

**Therapeutics.**—Jackson[8] describes the use of electrolysis in the treatment of diseases of the skin, especially with regard to hypertrichosis; in these cases the strength of the current should be one and a-half or two milliampères. The part should be aseptised before commencing, and the hairs should not be taken out close to one another, and four days' interval should always elapse before the same part is attacked again; the needle should never be entered twice into the same hair follicle; if these precautions are taken the resulting scars are insignificant. The use of electrolysis in nævi is also described, and in some cases he uses a tenotomy knife connected with the negative pole of the battery; this knife is also useful for breaking up fibromata and other small tumours. Although lupus nodules can be destroyed by electrolysis, this treatment is very tedious, and so is not recommended except for small nodules. Lastly, he finds electrolysis the best method of removing powder stains and tatoo marks—the needle is passed into each blue or black dot and kept in for about half a minute; this leaves a minute white cicatrix.

Skene[9] recommends the removal of small nævi, nævus pilaris, and papilloma by the galvano-cautery, and reserves electrolysis for vascular tumours where it is desirable to preserve the skin.

Levezier[10] recommends the treatment of impetigo by electrolysis. After its use there is a diminution of the itching, which is obtained often after the first sitting, but always after the second. The secretion ceases in a short time, and after the second or third sitting desquamation takes place, the crust dries up and falls off, and is not renewed. The erythema is also less, and the areola surrounding the impetiginous patch becomes pale. The hair in the neighbourhood of the patches loses its dryness and becomes darker and oily. Lastly, the glandular enlargement quickly subsides.

Tousey[11] gives a further report on the use of thiosinamine in the treatment of inoperable tumours and cicatricial conditions. He uses a hypodermic solution, made by dissolving 10 parts of thiosinamine in 100 parts of sterilised mixture of water and glycerine. He injects 12 to 15 mm. as a full dose into the triceps or gluteal regions every three days. After the injections, the number of white corpuscles in the blood will be found to be diminished. He considers that the drug is free from deleterious effects of any kind, but too large or too frequent doses might cause nausea; carefully conducted, the treatment produces a tonic effect, and none of his cases of keloid have lost a day's work while under treatment. In a case of sarcoma of the bladder injections were followed by a relief of the symptoms. His cases of keloid remained free from recurrence. Willems[12] points out that picric acid is of great utility in burns of the first and second degrees. The special action of the acid is to favour the growth of the new epidermis; it also has a marked analgesic action. By its means extensive burns of the face and limbs have healed with great rapidity, and epidermisation takes place so rapidly that no suppuration occurs. In burns of the third degree the acid is less useful; it does indeed check suppuration, but has no effect in quickening granulation. In mixed burns the acid may be used at first, as it soothes the pain and rapidly heals the superficial lesions; an antiseptic can then be substituted for the treatment of the granulating surface. He uses the acid in vaseline ointment of the strength of 1 or, at most, 2 per cent.; 15 grains of this spread on lint makes a dressing suitable for a vast burn. He has seen no toxic effect even in children, and the pain has been transient. The sole drawback is the yellow discolouration caused by the application, but this can be got rid of by repeated washing with alcohol, or by carbonate of lithia diluted with water.

[6] Nouv. Remed., April 24, 1898, p. 176. [7] Sem. Med., Mar. 2, 1898. [8] Med. Rec., June 4, 1898, p. 798. [9] Amer. Med. Surg. Bull., Mar. 10, 1898, p. 205. [10] Nouv. Med., Dec. 15, 1897; Med. Rec., May 28, 1898. [11] Med. Chron., Mar., 1898, page 440; New York Med. Jour., Nov. 6, 1897. [12] Amer. dec a Soc. Belg. de Chirg., Ann. 6, No. 2, May 15, 1898; Epit. Brit. Med. Jour., May 28, 1898.

